# Anticonvulsant effects of isopimpinellin and its interactions with classic antiseizure medications and borneol in the mouse tonic–clonic seizure model: an isobolographic transformation

**DOI:** 10.1007/s43440-023-00532-x

**Published:** 2023-10-11

**Authors:** Jarogniew J. Łuszczki, Hubert Bojar, Katarzyna Jankiewicz, Magdalena Florek-Łuszczki, Jarosław Chmielewski, Krystyna Skalicka-Woźniak

**Affiliations:** 1 Department of Occupational Medicine, Medical University of Lublin, 20-090 Lublin, Poland; 2 Department of Toxicology and Food Safety, Institute of Rural Health, 20-950 Lublin, Poland; 3 2nd Department of Gynecology, Medical University of Lublin, 20-954 Lublin, Poland; 4 Department of Anthropology, Institute of Rural Health, 20-950 Lublin, Poland; 5 Institute of Environmental Protection, National Research Institute, 02-170 Warsaw, Poland; 6 Department of Natural Products Chemistry, Medical University of Lublin, 20-093 Lublin, Poland

**Keywords:** Isopimpinellin, Coumarin, Borneol, Antiseizure medication, Isobolographic transformation, Maximal electroshock

## Abstract

**Background:**

Overwhelming evidence indicates that some naturally occurring coumarins and terpenes are widely used in folk medicine due to their various therapeutic effects affecting the brain. Antiseizure medications (ASMs) are the principal treatment option for epilepsy patients, although some novel strategies based on naturally occurring substances are intensively investigated. This study was aimed at determining the influence of isopimpinellin (ISOP—a coumarin) when administered either separately or in combination with borneol (BOR—a monoterpenoid), on the antiseizure potencies of four classic ASMs (carbamazepine (CBZ), phenytoin (PHT), phenobarbital (PB), and valproate (VPA)) in the mouse model of maximal electroshock-induced (MES) tonic–clonic seizures.

**Materials:**

Tonic–clonic seizures were evoked experimentally in mice after systemic (*ip*) administration of the respective doses of ISOP, BOR, and classic ASMs. Interactions for two-drug (ISOP + a classic ASM) and three-drug (ISOP + BOR + a classic ASM) mixtures were assessed isobolographically in the mouse MES model.

**Results:**

ISOP (administered alone) had no impact on the anticonvulsant potencies of four classic ASMs. Due to the isobolographic transformation of data, the combination of ISOP + VPA exerted an antagonistic interaction, whereas the two-drug mixtures of ISOP + CBZ, ISOP + PHT, and ISOP + PB produced additive interactions in the mouse MES model. The three-drug combinations of ISOP + BOR with CBZ and PHT produced additive interactions, while the three-drug combinations of ISOP + BOR with PB and VPA exerted synergistic interactions in the mouse MES model.

**Conclusions:**

The most intriguing interaction was that for ISOP + VPA, for which the addition of BOR evoked a transition from antagonism to synergy in the mouse MES model.

## Introduction

Epilepsy as a chronic neurological disorder affects approximately 1% of the world’s population [1, 2]. The principal treatment option for people suffering from epilepsy is monotherapy with a current frontline antiseizure medication (ASM) [3]. If monotherapy fails twice, the patients are on adjunctive treatment as a third option to control their seizures [4]. Despite 25 various ASMs available currently on the pharmaceutical market to treat convulsive and non-convulsive seizures, one-third of epilepsy patients is resistant to the standard ASMs and need more advanced treatment regimens (including dual and triple combinations of ASMs or curative application of novel compounds added to the standard treatment to enhance the anticonvulsant action of the ASMs) so as to properly manage their seizures [5, 6].

Relatively recently, scientific attention has been focused on traditional medicine and curative application of various herbs and medicinal plants in the treatment of patients with epilepsy, due to the confirmed anticonvulsant effects of botanical drugs. The best example of a naturally occurring compound used for ages in traditional medicine in the treatment of epilepsy, which lately became an ASM is cannabidiol (under the tradename of Epidiolex^®^) [7]. Of course, in traditional medicine, cannabidiol was not used alone as a pure unique drug, but it was given in a mixture of various phytocannabinoids (plant-origin cannabinoids) extracted from leaves of *Cannabis sativa* spp. There is no doubt that millennial experience in the curative application of some specific herbs and medicinal plants in patients with epilepsy [8, 9], prompted researchers to search for the most active naturally occurring compounds capable of terminating convulsive attacks and/or alleviating seizures [10]. Due to ultra-modern analytical techniques and innovative in vivo assays, it is possible to quickly identify and isolate the bioactive compounds responsible for the anticonvulsant properties of herbal remedies used in traditional medicine [11–13]. For instance, some *Cannabis sativa* L. extracts containing the active ingredients, including Δ9-tetrahydrocannabinol, cannabidiol, and various terpenes (identified qualitatively and quantitatively by ultra-HPLC technique), were investigated with respect to their anticonvulsant properties in an experimental seizure model in mice [14].

At present, a number of botanical drugs containing coumarins are used in traditional medicine for their stomachic, spasmolytic, and sedative effects [15–17]. Accumulating evidence indicates that various naturally occurring coumarins (including osthole (OST), imperatorin (IMP), xanthotoxin (XNT), umbelliferone (UMB), and scoparone (SCP)) exerted the anticonvulsant properties in the mouse maximal electroshock-induced seizure (MES) model [18–24]. Additionally, the mentioned coumarins enhanced the anticonvulsant potencies of some classic ASMs in the mouse MES model [18–24]. For instance, UMB and SCP significantly potentiated the anticonvulsant action of phenobarbital (PB) and valproate (VPA), but not that of phenytoin (PHT) and carbamazepine (CBZ) [23, 24]. IMP potentiated the antiseizure potencies of CBZ, PHT, and PB, but not that of VPA [21]. XNT enhanced the anticonvulsant action of CBZ and VPA, but not that of PHT and PB [25]. In contrast, OST had no impact on the antiseizure potencies of CBZ, PHT, PB, and VPA [20, 26]. It is worth noting that the MES test in mice corresponds to tonic–clonic seizures and, to a certain extent, to partial convulsions with or without secondary generalization in humans [27].

Accumulating experimental evidence indicates that borneol (BOR—a monoterpenoid) promotes the accumulation of other drugs in brain tissue and increases the brain bioavailability of drugs, including OST [28], and some classic ASMs [24]. BOR inhibits the activity of P-gp in the brain microvascular endothelial cells and reduces the expression of multidrug resistance proteins Mdr1a, Mdr1b, and Mrp1 [29, 30]. Additionally, in the anticonvulsant screening test, BOR itself protected the animals against tonic–clonic seizures in mice subjected to the MES test [31].

In this study, we intended to determine the anticonvulsant profile of isopimpinellin (ISOP)—another naturally occurring coumarin, whose molecular structure is closely related to XNT—a coumarin with confirmed anticonvulsant properties [32]. In the anticonvulsant screening test, ISOP (when administered alone) protected the mice against MES-induced seizures [33]. Additionally, this study was aimed at determining the impact of ISOP (administered alone or in combination with BOR) on the anticonvulsant potencies of four classic ASMs (CBZ, PHT, PB, and VPA) in the mouse model of tonic–clonic seizures in mice.

## Materials and methods

### Animals and experimental conditions

Male CD-1 outbred mice (8–9 weeks old) were used in this study. Laboratory conditions before and during the experiments were in strict accordance with the EU Directive 2010/63/EU for animal experiments and complied with the ARRIVE guidelines. The animals were housed in a specific pathogen-free facility with a controlled environment and with free access to tap water and food (ad libitum), under standardized housing and laboratory conditions (for more details see [34]). After a week adaptation to laboratory conditions, the mice (weighing 24 ± 3 g) were randomly assigned to experimental groups comprising 8 mice per group. All efforts were made to refine procedures, protect the animals’ welfare, minimize animals’ suffering, and use only the number of animals necessary to produce reliable scientific data according to the 3Rs rule. The experimental protocols and procedures described below were approved by the Local Ethics Committee at the University of Life Sciences in Lublin (License no.: 23/2018 from the 12th February 2018). Total number of mice used in this study was 256.

### Drugs

BOR, carbamazepine (CBZ), phenytoin (PHT), phenobarbital (PB), and valproate (VPA) were purchased from Sigma-Aldrich (St. Louis, MO, USA). VPA was dissolved in distilled water while the remaining studied drugs (i.e., BOR, ISOP, CBZ, PHT, and PB) were suspended in a 1% aqueous solution of Tween 80 (Sigma-Aldrich, St. Louis, MO, USA). All the drugs were administered intraperitoneally (ip) as follows: PHT—120 min; PB—60 min; CBZ, ISOP, and VPA—30 min, and BOR—15 min. before the experiments, as reported earlier [24]. ISOP was isolated from dichloromethane extracts obtained from fruits of *Pastinaca sativa* L. (Apiaceae). From 50 g of dried fruits 7.03 g of crude extract was obtained. The extraction and isolation procedure was similar to this described previously when high-performance counter-current chromatography was used to separate XNT for further ASM studies [35]. Briefly, the 1:1:1:1 n-heptane–ethyl acetate–methanol–water system was used in a reversed-phase system. A portion of the crude extract (200 mg) was dissolved in the biphasic solvent system (6 mL) and injected into a preparative coil of HPCCC Spectrum model (Dynamic Extractions, Slough, UK) multilayer coil-planet J-type centrifuge. The flow rate of the mobile phase was 6 mL/min. Fractions were collected every one minute, then evaporated under reduced pressure, and dissolved in methanol for subsequent purity analysis by HPLC–DAD. The yield of purified ISOP from the injection of crude extract (200 mg) was 3.6 mg with purity of 98%. All of the organic solvents used for extraction and isolation of ISOP were obtained from the Polish Chemical Reagent (Gliwice, Poland) and were of analytical reagent grade. Methanol for HPLC was of chromatographic grade (J.T. Baker Inc., Phillipsburg, NJ, USA), and water used was purified using a Millipore laboratory ultrapure water system (Simplicity TM system, Millipore, Molsheim, France). The standard of ISOP was obtained from Biopurify Phytochemicals Ltd. (Chengdu, Sichuan, China).

### Maximal electroshock-induced tonic–clonic seizures in animals

Electroconvulsions (tonic–clonic seizures) in mice were evoked by an alternating current (50 Hz; 500 V; 25 mA) delivered via auricular electrodes. The increasing doses of the classic ASMs were administered and after the respective pretreatment times, the animals were subjected to electroconvulsions. The percent of mice without any seizure activity (i.e., protected from tonic–clonic seizures) along with the respective doses of the ASMs was computed and presented as the median effective dose (ED_50_) of the ASMs, based on the log-probit method [36]. Each ED_50_ value corresponds to a dose of the ASM (in mg/kg) predicted to protect half of the animals tested in the MES test. In this study, ISOP was administered in constant doses of 25 and 50 mg/kg; BOR—in a fixed dose of 25 mg/kg; CBZ and PHT—at doses ranging between 6 and 16 mg/kg, PB—at doses ranging between 15 and 35 mg/kg and VPA—at doses ranging between 100 and 350 mg/kg.

### Isobolographic transformation of interactions

Doses of all the studied drugs in the mixture (i.e., BOR, ISOP, and classic ASMs) were transformed to the fractions of their ED_50_ values (from the MES test) and subsequently underwent the isobolographic transformation, as described earlier [37]. Due to the summation of the fractions of all the drugs in the mixture, it was possible to isobolographically characterize the interactions among the tested drugs such as additive, supra-additive (synergistic), or sub-additive (antagonistic) in the mouse MES model. The constant doses of ISOP in the mixture were illustrated graphically as parallel lines to the Y-axis, reflecting these doses, whereas the increasing doses of the ASMs allowed creating the isoboles, as reported earlier [24]. Such a type of transformation takes into account doses of all drugs present in the mixture and their anticonvulsant effects [37].

### Statistical analysis

The ED_50_ values (± SEM) for classic ASMs were calculated from the log-probit method [36], and the method transforming 95% confidence limits to SEM, as described in detail earlier [38]. Statistical comparisons of the respective ED_50_ values were performed with a one-way ANOVA test followed by the Dunnett’s post hoc test. Statistical comparisons of the respective isobolographic ED_50exp_ and ED_50add_ values for each combination were performed with the unpaired Student’s t-test. GraphPad Prism (version 8.0, San Diego, CA, USA) was used for statistical analysis of all the data (from the MES test and isobolographic transformation). All the isobolograms were drawn in the MS Excel spreadsheet. Differences among the compared values were considered statistically significant if p < 0.05.

## Results

### Influence of isopimpinellin on the anticonvulsant potency of four classic ASMs in the mouse MES model

**Fig. 1:**
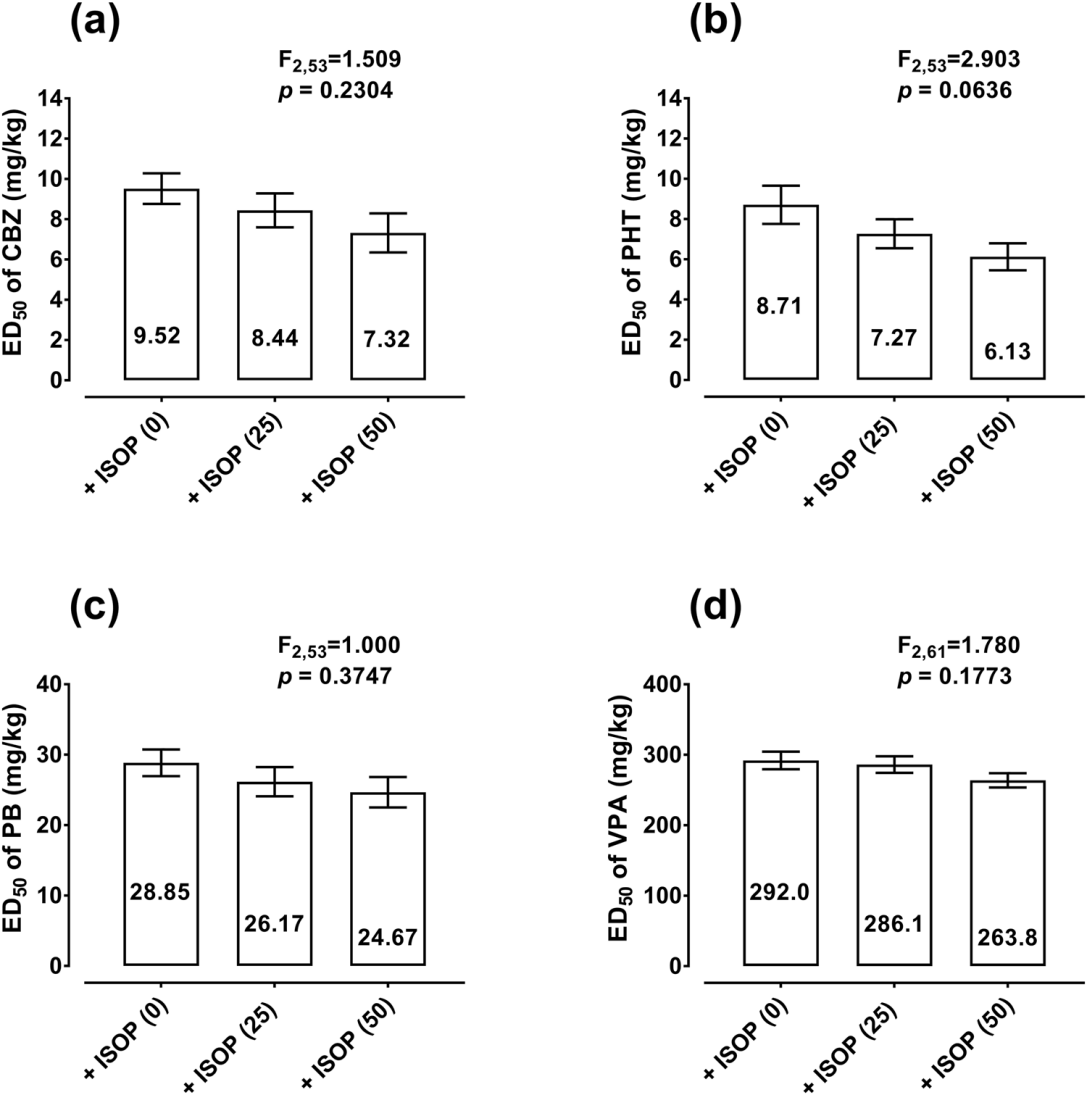
Effects of ISOP on the anticonvulsant potency of four classic ASMs in the mouse tonic–clonic seizure model. The columns indicate the ED_50_ values for CBZ (**a**), PHT (**b**), PB (**c**), and VPA (**d**) in the mouse MES model. *CBZ* carbamazepine, *ISOP* isopimpinellin, *PHT* phenytoin, *PB* phenobarbital, *VPA* valproate. Statistical analysis of data was performed with one-way ANOVA followed by Dunnett’s post hoc test. The total number of animals used for the calculation of ED_50_ values in this experiment was 192

ISOP administered *ip* at fixed doses of 25 and 50 mg/kg had no impact on the antiseizure effects of CBZ, PHT, PB, and VPA in the mouse MES model (Fig. 1). In the case of CBZ, ISOP at 25 and 50 mg/kg slightly reduced the ED_50_ value of CBZ in the mouse MES model from 9.52 mg/kg to 8.44 mg/kg (by 11%) and 7.32 mg/kg (by 23%), respectively (Fig. 1a). However, no significant changes among the ED_50_ values for CBZ were revealed with a one-way ANOVA test (Fig. 1a). ISOP at 25 and 50 mg/kg also reduced the ED_50_ value of PHT from 8.71 mg/kg to 7.27 mg/kg (by 17%) and 6.13 mg/kg (by 30%), respectively (Fig. 1b), but no significance was observed with a one-way ANOVA test (Fig. 1b). Similarly, ISOP at 25 and 50 mg/kg diminished the ED_50_ value of PB from 28.85 mg/kg to 26.17 mg/kg (by 9%) and 24.67 mg/kg (by 14%), respectively (Fig. 1c). ISOP administered *ip* at doses of 25 and 50 mg/kg decreased the ED_50_ value of VPA from 292 mg/kg to 286 mg/kg (by 2%) and 264 mg/kg (by 10%), respectively (Fig. 1d). In both cases, differences among the ED_50_ values for PB and VPA were insignificant with a one-way ANOVA test (Fig. 1c and d). ISOP when administered alone protected, in a dose-dependent manner, the animals from MES-induced tonic–clonic seizures, and its ED_50_ value amounted to 235.7 ± 23.5 mg/kg (results not shown).

### Isobolographic transformation of interaction between isopimpinellin and four classic ASMs in the mouse MES model

**Fig. 2:**
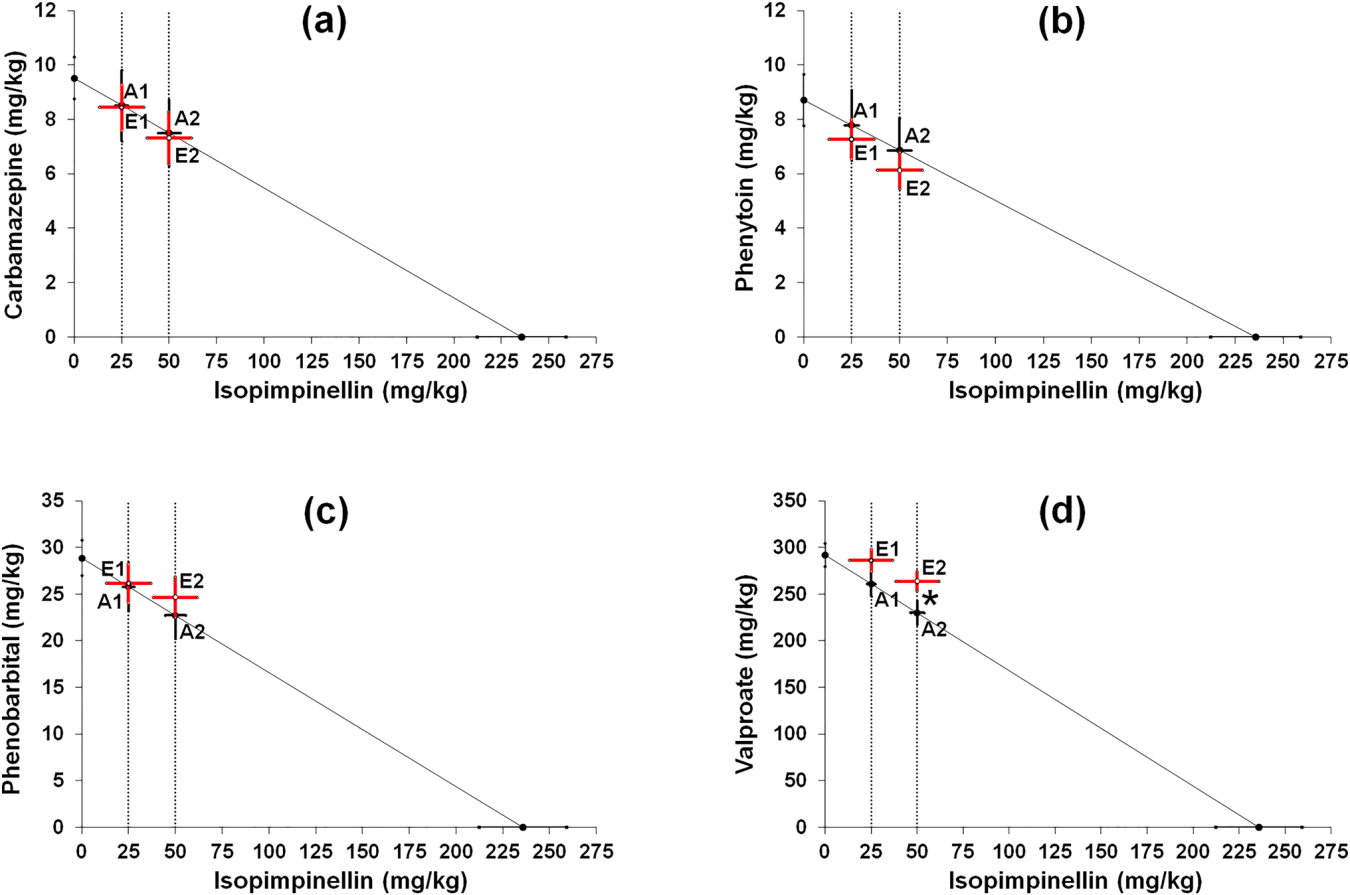
Isobolographic transformation of interactions between ISOP and four classic ASMs in the mouse MES model. Interactions between isopimpinellin and carbamazepine (**a**), phenytoin (**b**), phenobarbital (**c**), and valproate (**d**) are plotted graphically on the system of coordinates. The constant doses of isopimpinellin (25 and 50 mg/kg) in mixtures are plotted as parallel dotted lines to the Y-axis. The diagonal line on each diagram, connecting both X- and Y-axes is the line of additivity, which reflects the theoretically calculated ED_50add_ values. Intersections of the diagonal line with parallel dotted lines illustrate points of additivity (A1 and A2) that correspond to the theoretically calculated ED_50add_ values. Points E1 and E2 reflect the experimentally derived ED_50exp_ values for the two-drug mixtures (ISOP + ASM). *p < 0.05 vs. the respective ED_50add_ value (Student’s *t* test with Welch’s correction)

The ED_50_ values of four classic ASMs (CBZ, PHT, PB, and VPA) along with constant doses of ISOP (25 and 50 mg/kg) were transformed isobolographically so as to classify the exact types of interactions for the studied two-drug combinations (Table 1; Fig. 2a–d). In the case of CBZ, PHT, and PB, no significant difference was observed between the respective ED_50exp_ and ED_50add_ values, confirming the additive nature of interactions among the tested classic ASMs with ISOP (Table 1; Fig. 2a–c). In contrast, the ED_50exp_ value for the mixture of VPA with ISOP (50 mg/kg) considerably differed from the corresponding ED_50add_ value (*p < 0.05; Table 1; Fig. 2d), confirming a sub-additive (antagonistic) interaction between drugs in the mouse MES model (Table 1; Fig. 2d). With isobolographic transformation, it was found that ISOP (25 mg/kg) when combined with VPA produced additive interactions in the mouse MES model (Table 1; Fig. 2d).

**Table 1:** Isobolographic transformation of the anticonvulsant potency of ISOP and four classic ASMs in the mouse MES model

Combination	ED_50exp_	*n*_exp_	ED_50add_	*n*_add_	*t* test statistics	Sum of fractions
CBZ + ISOP (25)	33.44 ± 0.84	16	33.51 ± 2.33	44	*t*_52_ = 0.028, *p* = 0.978	0.86 + 0.11 = 0.97
CBZ + ISOP (50)	57.32 ± 0.97	24	57.50 ± 3.54	44	*t*_49_ = 0.049, *p* = 0.961	0.77 + 0.21 = 0.98
PHT + ISOP (25)	32.27 ± 0.72	16	32.79 ± 2.42	44	*t*_50_ = 0.206, *p* = 0.838	0.83 + 0.11 = 0.94
PHT + ISOP (50)	56.13 ± 0.67	24	56.86 ± 3.61	44	*t*_46_ = 0.199, *p* = 0.843	0.70 + 0.21 = 0.91
PB + ISOP (25)	51.17 ± 2.07	16	50.79 ± 2.84	44	*t*_56_ = 0.108, *p* = 0.914	0.91 + 0.11 = 1.02
PB + ISOP (50)	74.67 ± 2.16	24	72.73 ± 3.99	44	*t*_62_ = 0.428, *p* = 0.670	0.86 + 0.21 = 1.07
VPA + ISOP (25)	311.13 ± 11.73	24	286.00 ± 7.62	52	*t*_43_ = 1.797, *p* = 0.079	0.98 + 0.11 = 1.09
VPA + ISOP (50)	313.8 ± 10.15*	32	280.0 ± 8.19	52	*t*_67_ = 2.589, *p* = 0.012	0.90 + 0.21 = 1.11

Results are presented as ED_50exp_ and ED_50add_ values (in mg/kg ± SEM) of the classic ASMs in combination with ISOP in the mixture

*n* number of animals at those doses whose anticonvulsant effects ranged between 4th and 6th probit

^*^p < 0.05 vs. the respective ED_50add_ value (Student’s *t* test with Welch’s correction)

### Influence of isopimpinellin (ISOP) in combination with borneol (BOR) on the anticonvulsant action of four classic ASMs in the mouse MES model

**Fig. 3:**
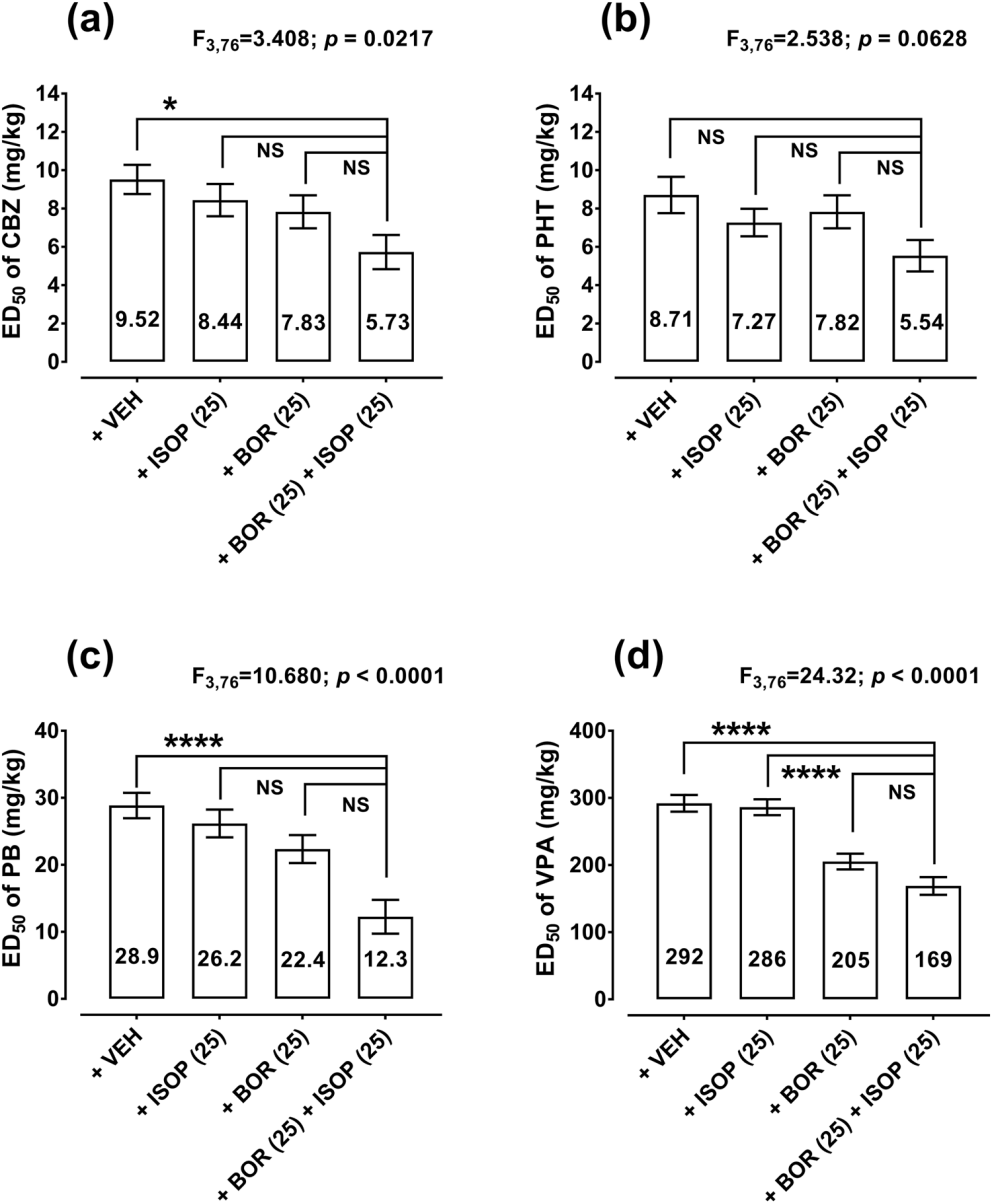
Effects of isopimpinellin (ISOP) in combination with borneol (BOR) on the anticonvulsant potency of four classic ASMs in the mouse tonic–clonic seizure model. The columns indicate the approx. ED_50_ values for CBZ (**a**), PHT (**b**), PB (**c**), and VPA (**d**) in the mouse MES model. *CBZ* carbamazepine, *PHT* phenytoin, *PB* phenobarbital, *VEH* vehicle, *VPA* valproate. *p < 0.05 and ****p < 0.0001 vs VEH-treated (control) animals (one-way ANOVA followed by Dunnett’s post hoc test). The total number of animals used for the calculation of ED_50_ values in this experiment was 256

In the case of CBZ, the combination of ISOP (25 mg/kg) + BOR (25 mg/kg) significantly reduced (by 40%) the ED_50_ value of CBZ in the mouse MES model from 9.52 mg/kg to 5.73 mg/kg (*p < 0.05; Fig. 3a). The combination of ISOP (25 mg/kg) + BOR (25 mg/kg) also reduced the ED_50_ value of PHT (by 36%) from 8.71 mg/kg to 5.54 mg/kg (Fig. 3b), but no significance was observed with a one-way ANOVA test (Fig. 3b). In contrast, ISOP (25 mg/kg) + BOR (25 mg/kg) considerably diminished the ED_50_ value of PB (by 58%) from 28.9 mg/kg to 12.3 mg/kg (****p < 0.0001; Fig. 3c). Similarly, ISOP (25 mg/kg) + BOR (25 mg/kg) significantly decreased the ED_50_ value of VPA (by 42%) from 292 mg/kg to 169 mg/kg (****p < 0.0001; Fig. 3d).

### Isobolographic transformation of interactions for three-drug combinations among borneol (BOR), isopimpinellin (ISOP), and four classic ASMs in the mouse MES model

The three-drug combination of CBZ with ISOP (25 mg/kg) + BOR (25 mg/kg) exerted additive interaction in the mouse MES model with the fraction index value of 0.81 (Table 2). Similarly, the combination of PHT with the constant doses of ISOP and BOR produced additive interaction in the mouse MES model with the fraction index value amounting to 0.85 (Table 2). In the case of the combination of PB with ISOP + BOR, the experimentally derived ED_50exp_ values significantly differed from the additively calculated ED_50add_ values (*p < 0.05), indicating the synergistic interaction among the studied three drugs (Table 2). For the combination of VPA with ISOP + BOR, the synergistic interaction was observed in the mouse MES model because the experimentally derived ED_50exp_ values significantly differed from the additively calculated ED_50add_ values (***p < 0.001; Table 2). For the synergistic interactions, the fraction index values amounted to 0.63 for the combination of PB with ISOP + BOR, and 0.79 for the combination of VPA with ISOP + BOR, respectively (Table 2).

**Table 2:** Isobolographic transformation of the anticonvulsant potency of three-drug combination—ISOP, BOR, and four classic ASMs in the mouse MES model

Combination	ED_50exp_	*n*_exp_	ED_50add_	n_add_	*t* test statistics	Sum of fractions
CBZ + ISOP (25) + BOR (25)	55.73 ± 0.89	24	57.50 ± 3.54	44	*t*_48_ = 0.485, *p* = 0.630	0.60 + 0.11 + 0.10 = 0.81
PHT + ISOP (25) + BOR (25)	55.54 ± 0.82	24	56.86 ± 3.61	44	*t*_47_ = 0.357, *p* = 0.723	0.64 + 0.11 + 0.10 = 0.85
PB + ISOP (25) + BOR (25)	62.25 ± 2.52 *	24	72.73 ± 3.99	44	*t*_65_ = 2.221, *p* = 0.030	0.42 + 0.11 + 0.10 = 0.63
VPA + ISOP (25) + BOR (25)	218.9 ± 13.15 ***	24	280.0 ± 8.19	52	*t*_41_ = 3.944, *p* = 0.0003	0.58 + 0.11 + 0.10 = 0.79

Results are presented as ED_50exp_ and ED_50add_ values (in mg/kg ± SEM) of the classic ASMs in combination with ISOP and BOR

*n* number of animals at those doses whose anticonvulsant effects ranged between 4th and 6th probit

^*^p < 0.05 and

^***^p < 0.001 vs. the respective ED_50add_ value (student’s *t* test with Welch’s correction)

## Discussion

The anticonvulsant effects observed for ISOP in combinations with four classic ASMs are quite similar to those reported earlier for osthole (OST). More specifically, OST (simply coumarin) did not affect the anticonvulsant action of CBZ, PHT, PB, or VPA in the mouse MES model [20, 26]. Considering the structural formulas of ISOP, OST, and other coumarins, it is difficult to unequivocally predict the effects of these coumarins on the anticonvulsant activity of classic ASMs. In spite of the structural similarities of ISOP and XNT, their impact on classic ASMs is different. Inversely, the effects produced by ISOP when combined with classic ASMs are identical to those observed for OST, although their structural formulas are not similar. It is worth mentioning that the effects observed for UMB and SCP when combined with classic ASMs are identical (both coumarins potentiated the antiseizure effects of PB and VPA, but not those of CBZ or PHT), and their structural formulas are also quite similar. This fact may suggest the existence of a relationship between the structure of these coumarins (UMB and SCP) and their influence on classic ASMs in the mouse MES model. Unfortunately, such a relationship cannot be ascribed to ISOP and XNT, because the coumarins exerted different effects when combined with classic ASMs in the mouse MES model, albeit their chemical structures are quite similar.

On the other hand, the isobolographic transformation of data revealed that the combinations of ISOP with CBZ, PHT, and PB exerted additive interactions, but the combination of ISOP with VPA produced antagonistic interaction in the mouse MES model. From a pharmacological viewpoint, the antagonistic interaction in terms of seizure suppression is not favorable because ISOP reduced the anticonvulsant potency of VPA in mice subjected to the MES test, as compared to the control (VPA alone-treated) animals. In the case of the combinations of ISOP with CBZ, PHT, and PB, the additive interactions for these two drug mixtures can offer beneficial or neutral effects in terms of seizure suppression in experimental animals. Of note, the isobolographic additivity can be sometimes favorable, especially, if the two-drug mixture offers not only satisfactory seizure suppression but also lesser toxicity, as reported when the drugs are used separately and in higher doses than those when combined in the mixture (for more details, see [39, 40]).

No doubt exists that the isobolographic transformation of data is the best method used in applied pharmacology to classify interactions between drugs, especially, if one of the tested drugs or compounds is not an ASM, but it possesses the anticonvulsant potential [41]. The antagonistic interaction between ISOP and VPA, reported after the isobolographic transformation of data in the mouse MES model, prompted us not to recommend this combination for clinical conditions. A special warning about a potential antagonistic interaction that may occur when combining ISOP with VPA should be addressed to clinicians and potential patients to avoid some potentially harmful effects (i.e., reduction in seizure control) in further polytherapy.

Additionally, we evaluated in this study the influence of BOR in combination with ISOP on the anticonvulsant action of 4 classic ASMs in the mouse MES model. Previously, the effects of BOR (when administered alone) on the anticonvulsant potential of four classic ASMs were assessed in the mouse MES model [24]. Experimental evidence indicated that BOR significantly enhanced the antiseizure effects of PB and VPA in the mouse MES model but, the observed interactions resulted from pharmacokinetic increases in ASMs’ content in the brains of experimental animals [24]. It was reported that BOR significantly elevated total brain contents of PB and VPA, and this fact was the main reason for the significant impact of BOR on the anticonvulsant potentials of classic ASMs in the mouse MES model. Similarly, in this study, we determined the effects of the combinations of ISOP with BOR on the anticonvulsant potential of ASMs. The two-drug mixture of ISOP + BOR did not significantly affect the antiseizure effects of CBZ and PHT, but the mixture of ISOP + BOR enhanced the antiseizure effects of PB and VPA in the mouse MES model.

The isobolographic transformation of data for the three-drug combinations of ISOP + BOR + VPA and ISOP + BOR + PB revealed that the interactions were supra-additive (synergistic) in the mouse MES model, whereas the combinations of ISOP + BOR with CBZ and PHT were additive in nature. It was reported herein that BOR potentiated by itself the anticonvulsant potency of VPA in the mouse MES model, without any collaborative effects of ISOP. In contrast, BOR enhanced the anticonvulsant potency of PB in the mouse MES model offering the synergistic interaction, but only in the presence of ISOP. The lack of ISOP in the mixture makes the combination of BOR with PB only additive in the mouse tonic–clonic seizure model [24], despite an increase in the total brain content of PB after BOR administration.

Of note, a transition from antagonism to synergistic interaction has been demonstrated in this study in the mouse MES model in the case of the combination of ISOP + VPA. BOR seems to be a good candidate drug to increase brain permeability of some classic ASMs, especially PB and VPA.

The superiority of the isobolographic transformation of data over the subthreshold method used in experimental epileptology has been confirmed earlier [42]. Additionally, the isobolographic summation of the anticonvulsant fractions of all the tested drugs in the mixture makes the results more reliable (for more detailed information, see [37, 43]).

Another fact needs a special explanation when extrapolating the results from this preclinical study to further experimental conditions. The anticonvulsant effects produced by the mixture of ISOP + BOR on VPA were substantially higher than those observed for the mixture of VPA with ISOP alone. Adding BOR allowed for exceeding the antagonistic interaction for ISOP + VPA and exerting supra-additive interaction for the three-drug combination of ISOP + BOR + VPA in the mouse MES model. Although, the change in type of interaction from antagonism to synergy was highly likely associated with a significant elevation of total brain VPA concentrations (as reported earlier), this pharmacokinetic nature of interaction among drugs seems to be solely responsible for the observed effects.

This study has also some limitations, which should be mentioned herein, i.e., when translating or extrapolating the results of this study to clinical conditions. First, only some possible pharmacokinetic interactions among BOR and PB or VPA were taken into account because, as reported earlier, BOR elevated the total brain content of PB and VPA in experimental animals. However, neither the total brain content of ISOP and BOR in mice receiving ASMs was verified, nor total brain concentrations of CBZ and PHT were measured. Second, only one animal species (mice) was used. No experiments on rats or zebrafish larvae were conducted to confirm the anticonvulsant activity of ISOP in in vivo studies. Third, only one experimental seizure model (MES test) in mice was used as a model of tonic–clonic seizures. Fourth, the most important limitation is the fact that ISOP and BOR, in contrast to clinical conditions, were tested after their single systemic (*ip*) administration in the acute seizure model. No chronic (prolonged) oral administration of the tested substances and ASMs was performed. Despite all the above-mentioned limitations, this experimental study provides us with clear-cut information about the anticonvulsant activity of ISOP when administered alone and in combination with classic ASMs and BOR that would be potentially useful in further preclinical or clinical studies.

## Conclusions

ISOP when added to the studied classic ASMs (CBZ, PB, and PHT) produced additive interactions, but special attention should be paid to the combination with VPA, which occurred antagonistically in the mouse MES model. BOR added to the mixtures of ISOP with ASMs potentiated the anticonvulsant activity of PB and VPA, exerting synergistic interactions in the mouse MES model. The three-drug mixtures of BOR, ISOP, and CBZ or PHT produced additive interactions in the tonic–clonic seizure model in mice. Unfortunately, BOR significantly increased the total brain concentrations of the tested ASMs, contributing to the pharmacokinetic nature of observed synergistic interactions with VPA and PB in the mouse MES model. BOR in two-drug and three-drug mixtures with CBZ or PHT exerted additive interaction in the mouse MES model.

## Data Availability

The datasets generated during and/or analyzed during the current study are available from the corresponding author upon reasonable request.

## Abbreviations

ASM: Antiseizure medication
BOR: Borneol
CBZ: Carbamazepine
IMP: Imperatorin
ISOP: Isopimpinellin
MES: Maximal electroshock-induced seizures
OST: Osthole
PB: Phenobarbital
PHT: Phenytoin
SCP: Scoparone
UMB: Umbelliferone
VPA: Valproate
XNT: Xanthotoxin
